# Enhancement of sense of ownership using virtual and haptic feedback

**DOI:** 10.1038/s41598-024-55162-x

**Published:** 2024-03-01

**Authors:** Samirah Altukhaim, Daniel George, Kiruba Nagaratnam, Toshiyuki Kondo, Yoshikatsu Hayashi

**Affiliations:** 1 Biomedical Science and Biomedical Engineering, School of Biological Sciences, University of Reading, Whiteknights, Reading, RG6 6AY UK; 2 Physiotherapy Group in Stroke Unit, Alamiri Hospital, Kuwait, Kuwait; 3 Stroke Unit, Royal Berskhire Hospital, London Road, Reading, RG1 5AN UK; 4 Department of Computer and Information Sciences, Graduate School of Engineering, Tokyo University of Agriculture and Technology, 2-24-16, Naka-Cho, Koganei, Tokyo, Japan

**Keywords:** Sense of ownership, Virtual hand, Multimodal feedback, Haptic feedback, Synchronous, In phase and anti-phase, Cognitive neuroscience, Engineering

## Abstract

Accomplishing motor function requires multimodal information, such as visual and haptic feedback, which induces a sense of ownership (SoO) over one’s own body part. In this study, we developed a visual–haptic human machine interface that combines three different types of feedback (visual, haptic, and kinesthetic) in the context of passive hand-grasping motion and aimed to generate SoO over a virtual hand. We tested two conditions, both conditions the three set of feedback were synchronous, the first condition was in-phase, and the second condition was in antiphase. In both conditions, we utilized passive visual feedback (pre-recorded video of a real hand displayed), haptic feedback (balloon inflated and deflated), and kinesthetic feedback (finger movement following the balloon curvature). To quantify the SoO, the participants’ reaction time was measured in response to a sense of threat. We found that most participants had a shorter reaction time under anti-phase condition, indicating that synchronous anti-phase of the multimodal system was better than in-phase condition for inducing a SoO of the virtual hand. We conclude that stronger haptic feedback has a key role in the SoO in accordance with visual information. Because the virtual hand is closing and the high pressure from the balloon against the hand creates the sensation of grasping and closing the hand, it appeared as though the person was closing his/her hand at the perceptual level.

## Introduction

We perform most of our daily movements unconsciously without being fully mindful of how they are executed. Herein, the term “unconsciously” refers to the unconscious control over the movements of our hands without exerting any conscious and effort^1,2^. For example, voluntary movements become increasingly automatic with experience^3^, similar to reflexes^2^. As healthy individuals, we have mastered voluntary movements through repeated practice, and our unconscious proprioception now manages these complex actions.

Consider the daily action of drinking a cup of tea. Our eyes find and locate the cup (visual feedback) before we extend our forearm to reach for it. We open our hand by spreading our fingers away from the palm and then close it to hold the cup^4,5^. Finally, we ensure that our hand holds the cup using haptic feedback, which instructs the brain to locate and keep hold of it^6,7^. For all these movements to be rapid, precise, and coordinated, the nervous system must continuously receive and use sensory input from the outside world to adapt and correct the trajectory of our limbs.

Multimodal feedback streams combined with motor intention generate a sense of ownership (SoO) and a sense of agency (SoA)^8^. According to Gallagher^9^, SoO refers to the feeling of “mineness” or the conviction that an object inherently belongs to oneself. Whereas SoA is subjective awareness of initiating, executing, and controlling one’s own body movements, together with their external consequences^10^. According to Smith et al.^11^, stroke can impair a patient’s ability to regulate their upper limb muscles, which can disrupt both SoA and SoO^9^ specifically in the affected limb. In some cases, patients might neglect the impaired body part.

In a recent study by Aizu et al.^12^, the participants’ reaction times (RT) were utilized to assess body-specific attention in both healthy individuals and stroke patients. The findings suggested that a faster RT correlated with higher body-specific attention. Notably, post-stroke hemiparesis patients exhibited a time-dependent decline in attention towards the paretic hand. This decline implies a learned avoidance using the affected limb because it is perceived as less useful for daily tasks, as opposed to brain damage^12^. This indicates dis-embodiment of the paretic limb, which might be linked to reduced ownership of the paretic hand^13^. The same paradigm of RT was extended to measure the sense of embodiment toward the prosthetic foot of amputees^14^.

The important goal of neurorehabilitation when incorporating multiple types of feedback is to enhance SoO and SoA over the artificial device. This enables the patient to navigate their physical surroundings and re-establish a sense of “belonging” toward this limb^15^. The predominant paradigm of limb ownership is a rubber hand illusion (RHI)^16^, wherein a fake hand (resembling the subject hand) is set in front of the subject, while their real hand is covered and not visible. The examiner simulates stroking on both hands, either synchronously or asynchronously. An illusory SoO is induced over the rubber hand when the stroking is synchronous. Previously, SoO has been measured subjectively using various methods such a questionnaire^16^, as well as physiological measures skin conductance^17^, and temperature of the skin^18^, to assess the proprioceptive drift level towards the fake (dummy) hand in response to participant threat-related fear.

The experimental paradigm used in this study is like the RHI because our integrated system was developed to provide passive motion to the participants. Motor commands or intentions, which are necessary elements for SoA, were not generated, limiting our focus to SoO. During passive movement, afferent pathways transmit signals from skin receptors, muscle spindles, joint receptors and visual feedback to the brain, which conveys information related to body position, movement and tactile sensations. These pathways are involved in the SoO^19^, whereas reafferent feedback signals from proprioception, efferent or central motor signals, vision, intended action and previous thoughts to actions are required for SoA^20^.

Visual information is considered the fundamental means through which humans interact with their environment^21^. However, it is essential to note that individuals with visual impairments or disabilities may rely on alternative senses or modalities for interaction with and perception of the outside world.

Recently, Ito and Gomi^22^ investigated whether or not visual information influenced the passive contraction of muscles known as the stretch reflex. They manipulated visual cues through experimental series and reflex mechanisms during the unconscious body movements in response to visual or proprioceptive stimuli. People are subject to visual signs for passing judgment on distances and positions, and proprioception alone is insufficient for the body to contact a far-off body part with great accuracy. Ito and Gomi^22^ found that the mind utilizes the body’s representation containing visual contribution to direct the stretch reflex.

Visual feedback has also been found to be important for reducing phantom limb pain (PLP), a severe type of pain often felt by amputees in the lost body part as if it still exists^23^. Mirror box therapy, which uses vision, is one of the most useful nonpharmacological approaches to treat PLP. It creates an illusion that the missing limb is moving, as the person looks at the intact limb in a mirror while the amputated limb is hidden^24^. Therefore, PLP can be treated using neurorehabilitation methods, which are widely applied in medical training due to their simplicity.

Mirror treatment has also been used with virtual reality (VR), wherein the visual picture of lost limbs is introduced in a virtual environment. SoO can be altered by visual stimuli in which a participant feels a SoO toward a virtual hand when merged with VR^25^. This phenomenon is heightened when the real hand's movement corresponds to that of the visual avatar. The use of multimodal systems to treat PLP has resulted in significant pain reduction^26,27^.

Sano et al.^28^ demonstrated that a multimodal system in VR reinforces the reality of patient experiences by introducing visual, auditory and tactile feedback interactions between objects and the virtual arm. Their findings suggest that tactile feedback strengthens the pain-reducing effect of the task in the VR system.

Expanding upon the implications of tactile feedback, a focal point emerges on the significance of “haptic perception”, specifically defined as the process of perceiving physical objects through one’s hands (touch)^6,29^. It begins developing during early childhood and continues until adolescence. We use our hands to understand the world inside our span (haptic perception) and act upon it (handling objects). Flanagan and Johansson^6^ investigated whether or not the perception of an object’s quality is influenced by the properties of other objects or the method of handling the object. They found that handling greatly influences the perception of the weight and shape of an object. Furthermore, visual input about the physical features of the object might help determine the appropriate grip force.

This underscores the importance of multimodal sensory feedback, as vision alone significantly increased awareness or phantom control, and tactile input during phantom motor performance may have provided an additional sensory feedback substitution. Therefore, when the subject perceives a multisensory input (visual and haptic feedback) from external objects, their sense of limb ownership toward the virtual hand (i.e., ownership-driven embodiment) might be enhanced. A combination of sensory modalities (i.e., visuo-tactile)^30^ bolster the illusory sensation^31^.

A long period of training using a prosthetic foot, involving the integration of multimodal training via visual information and motor control, might induce subjective “embodiment” toward the assistive device. This embodiment results from lower limb amputation patients directing their attention towards the prosthetic foot, treating it as an integral part of their body. Hence, the inclusion of multimodal training emerges as a vital factor for boosting the efficacy of patients' rehabilitation^14^.

The earlier-discussed literature underscores the vital role of multimodal systems in heightening the SoO over artificial devices. Expanding on this notion, previous research in multimodal systems has primarily focused on comparing synchronous and asynchronous conditions, specifically examining the interplay of visual feedback with haptic or other inputs to elucidate their combined influence on the perception of ownership over virtual or simulated hands within virtual environments.

While research had established that synchronicity enhances the SoO and asynchrony diminishes it, there was a distinct lack of exploration into various types of synchrony. Therefore, in this research, we introduced a new form of synchrony—termed 'anti-phase' synchrony—where opposite movements occurred between two inputs while they were synchronized.

The aim of the present study was to enhance the SoO over virtual hand by applying the multi-modal stimulation. To achieve this, we designed and developed an integrated system using virtually enhanced haptic technology combining three different types of feedback: visual, haptic and kinesthetic feedback. We tested two conditions (in-phase and antiphase of the three set of feedback) to identify the optimal condition that best integrates multimodal information to create SoO.

## Analysis and statistics

**Figure 1:**
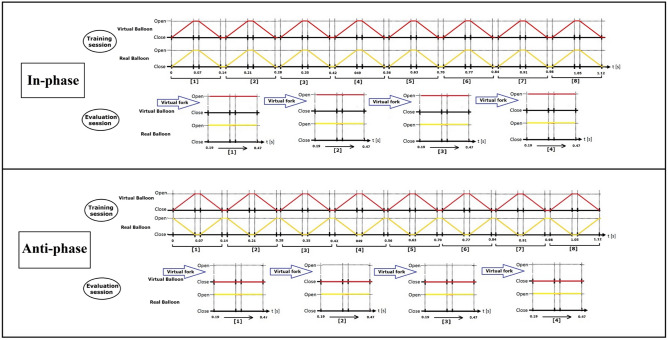
Details the conditions. Each condition during the training session and evaluation session. Under both conditions, eight inflation–deflation cycles were performed, each lasting 0.14 s starting from the deflated state. In the evaluation session, the virtual fork moved toward a static virtual hand from 0.19 s to 0.47 s. The reaction time was measured four times.

The measurement of the participants’ RTs was repeated four times in the evaluation session (Fig. 1). We calculated the median values of the RTs under each condition and compared it among participants.

The Shapiro–Wilk test was performed to determine whether samples were normally distributed. To ascertain if variation in response times of individual participants plays a role under the two conditions, and to determine the level of statistical significance, we performed the Wilcoxon signed rank test for non-parametric paired data.

We performed another Wilcoxon signed rank test on data from all 23 subjects together under each condition, neglecting the difference between participants, to establish the level of statistical significance of the distribution of RTs. Statistical significance was set at p = 0.05. All analyses were performed using the SPSS version 25 software.

## Results

**Figure 2:**
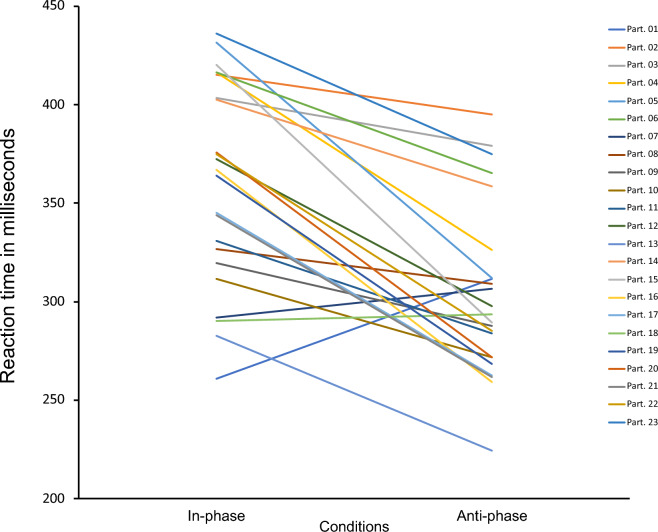
Line plot of the reaction time. We found that the participants’ median reaction times were consistently low under anti-phase condition for 20 out of 23 participants and consistently high under in-phase condition. Part. = Participant.

**Figure 3:**
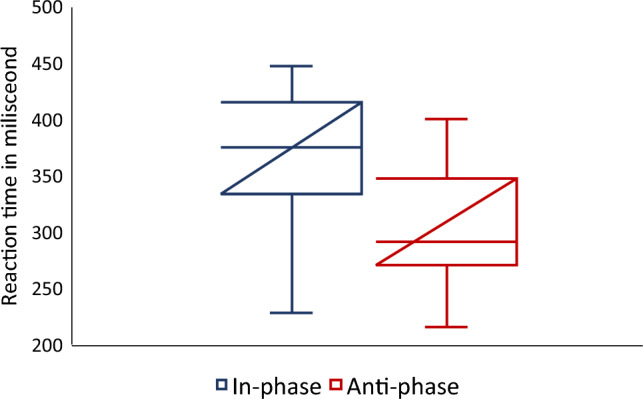
Boxplot of reaction times. The blue in the graph indicates in-phase condition, and the red colour indicates anti-phase condition.

The median value of RT for each participant under each condition was calculated to determine which condition could induce a higher SoO (shorter RT). The median RTs were consistently low under anti-phase condition in 20 out of 23 participants, whereas the median RTs of three participants (participants 1, 7 and 18) were consistently high under in-phase condition (Figs. 2 and 3). Each participant responded differently, with response times ranging from 195 to 470 ms. For example, the RT for participant 1 was 261 ms in in-phase condition and 331.5 ms in anti-phase condition. In contrast, the RT for participant 2 was 415 ms in in-phase condition and 395 ms in anti-condition. Our findings revealed that anti-phase condition could induce a higher SoO over the virtual hand.

Before the paired comparison analysis, we performed the Shapiro–Wilk test to check if the data met the normal distribution criteria. The resultant p-value of the normality test was mostly abnormal. For instance, participant 3 had p = 0.041 and p = 0.406 in in-phase and anti-phase conditions, respectively, whereas participant 5 had p = 0.590 and p = 0.027 in in-phase and anti-phase conditions, respectively. Since the RT data in some cases were not normally distributed, we used the Wilcoxon signed rank test instead to compare each participant’s RTs under the two conditions.

The RTs were significantly different between in-phase and anti-phase conditions for all participants, except numbers 7 (p = 0.09) and 18 (p = 0.456), whose RTs under anti-phase condition were high.

When we compared the data of 23 participants taken together between the two conditions, the RTs were found to be significantly different (p = 0.000143), confirming that anti-phase condition could induce a higher SoO over the virtual hand.

To subjectively evaluate the participants' response to the moving fork under the second condition anti-phase (The participants' responses in both conditions are available in the supplementary file), we asked the following questions: “To what extent do you think that you wanted to stop the fork from reaching your hand as fast as you could in the second condition?” Eight participants chose 4, and 12 participants chose 5 because they strongly agreed with this question. “To what extent do you think that you are threatened and stressed from the fork while it is moving toward the virtual hand in the second condition?” Eighteen participants chose 5 because they strongly agreed with this question. To evaluate the extent to which SoO was induced over the virtual hand under anti-phase condition, we asked the following: “To what extent do you think that the virtual hand belonged to your body (was part of your body) in the second condition?” Twenty subjects strongly agreed.

## Discussion

We found that the second condition, wherein visual, haptic and kinesthetic feedback were synchronous in “anti-phase”, induced a stronger SoO over the virtual hand on the basis of the measured RTs.

This result seems to be counterintuitive. Although the participants would not produce the motor intention of actively holding the balloon under the multimodal feedback provided passively to them, the visual feedback may trigger a sense of “active” grasping. Moreover, haptic feedback would play a key role as an augmentation factor to generate the sense of active grasping. Under “anti-phase” condition, although the kinesthetic factor opposes these two feedback streams (visual and haptics), the proprioceptive feedback might have a minor role in sensory integration^22^. According to the visual information provided to the participants during the evaluation phase, they see the virtual hand close on the display while the real balloon inflates, and their real hand opens. Consequently, we believe that applying high pressure to the skin with the balloon produces stronger haptic feedback to the skin, generating the perception that one is closing their hand. Although the anti-phase condition proprioceptive feedback (the opening of the hand) relays finger extension, it is expected to have a minor role relative to the roles visual and haptic feedback, i.e., be dominated by the visual and generated haptic sensations. In summary, the visual information and stronger haptic sensation of the closing hand may induce a feeling of active grasping by overwriting the kinesthetic information of opening the real hand. In the anti-phase condition, at the perceptual level, visual/haptic feedback was provided to the participants in an in-phase manner, while the kinesthetic feedback was provided in an anti-phase manner to the visual/haptic feedback.

Regarding the sense of active grasping, observing mirrored movement increases the activity of the motor network in the brain that is responsible for action observation, cognitive control, attention shift^32^ and reorganization of the sensorimotor cortex^33^. In this case, the concept of mirror therapy is triggered, as if one is performing the motion independently^34^.

Multiple studies have demonstrated the effectiveness of mirror therapy in neurorehabilitation to improve the motor function of stroke survivors^35,36^. It has also been shown to enhance the activity of those brain areas involved in self-awareness and spatial attention^36^. The visual feedback of the affected or amputated limb, which is observed from a first-person perspective (1PP), is accompanied by SoO for the reflected image, as the amputee perceives it as part of their own body^37^. On the other hand, in healthy subjects, the perception of a mirrored limb is related to activity in the superior parietal cortex, which represents a key function for the perception of body consciousness^38^. Hence, this can enhance SoO^39^.

In using visual information as the main type of feedback, our experiment is similar to mirror therapy. The participants watched the dynamic movement of a virtual hand on the display, and from the 1PP, this visual information replaced their real hand underneath the display. In mirror therapy, the mirrored hand is a reflection of the healthy hand but placed closer to the impaired hand. Extending traditional mirror therapy, we incorporated haptic feedback in our experiment, which plays a crucial role in enhancing SoO over the virtual hand under the anti-phase condition. In summary, visual information depicting the body part’s movements from a 1PP could activate the motor area of the brain^40^, as if the observer is performing the movement which induces SoO over the virtual hand because the participants feel like they are controlling the motion of the virtual hand simply by looking at it^41^.

Furthermore, in multimodal sensory binding, haptic technology works by integrating appropriate software adjustments with an appropriate physical sensation using the sense of touch. Haptic feedback transmits and comprehends information about physical situations^42^. Participants can run their fingers across a surface to derive an idea of its size and form^43^. Robles-De-La-Torre and Hayward^44^ performed an experiment that identified participants who used force signals unrelated to surface shape. A hump’s force signals combined with a hole’s geometry caused responders to perceive a bump, while a hole was perceived when force signals from a hole were combined with the geometry of a bump. In accordance with their findings, in the second condition of our experiment, the high pressure of the balloon could have caused the participants to sense the force as the hand closed, which was in in-phase with the visual feedback movements that occurred when the virtual hand closed.

From our findings, a shorter RT may have indicated higher SoO over the virtual hand, which is supported by the experiment on lower limb amputees^14^, wherein their attention was directed to the prosthetic foot using visual detection. This finding suggested that humans pay more attention to the body parts integrated into “embodiment” and respond faster in terms of sensing and reacting to the body parts.

We focused on the objective measurement of the SoO as opposed to the subjective measurement, such as a questionnaire, and believe that the measurement of RT would be more appropriate than the measurement of physiological reactions, as the primary function of the SoO would be embedded in the sensory-motor loops.

It is often considered that subjective measures, such as questionnaires, may have limitations in terms of their reliability and validity. Therefore, it is generally preferable to use objective measures, e.g., RT, because they provide more quantifiable and observable data. However, it is important to note that we do not prefer to entirely disregard subjective responses. Subjective responses can offer unique insights into individuals’ personal experiences, perceptions and emotions.

The responses to the questionnaire support our findings. One response stated, “When I spotted a moving fork, it felt like my heart rate was increasing, I wanted to [stop] it as soon as possible.” Another participant reported, “I felt the fork wanted to touch my hand and I didn’t stop because I wanted to feel it, but then I realised that it [was] not my real hand and only [was] a virtual hand.” These responses could indicate that a SoO of the virtual hand was induced.

While the experiment aimed to measure which condition might induce a higher sense of ownership over the virtual hand on the display, it is crucial to acknowledge potential side effects that could have influenced the outcomes. For example, order effects might impact participants' responses. The initiation of the first condition could have potentially trained participants, resulting in faster reactions during subsequent conditions. Therefore, for future studies, consideration might be given to switching the order of conditions to mitigate any order-related biases and enhance the robustness of the findings.

This study, with potential applications in clinical rehabilitation, suggests that initially training patients to enhance their SoO over a virtual hand using synchronized anti-phase condition may contribute to strengthening this sense as observed in our study. Following the induction of an enhanced SoO, patients can then begin sessions utilizing virtual enhanced haptic feedback in a virtual reality environment.

## Conclusions

In summary, this study has highlighted that the synchronous alignment of three feedback types (visual, kinesthetic, and haptic), particularly when in anti-phase coordination, may enhance a stronger SoO over virtual hand This was evidenced by the participants exhibiting shorter reaction times in the presence of a perceived threat.

At the perceptual level, participants may have perceived the feedback as follows: the Visual/Haptic feedback operated in-phase, while the Kinesthetic Feedback operated in anti-phase to the Visual/Haptic feedback. However, within the experimental paradigm, the movement of the virtual hand holding the balloon on the display was opposite to the movement of the real hand, which was also holding a balloon positioned underneath the display. The visual and haptic feedback seemed to have had a greater impact when synchronized in-phase. For instance, when the visual hand grasped an inflated balloon shown on the display, the real hand remained open due to the inflated balloon it held. It was possible that the pressure from the balloon against the hand played a crucial role in triggering the hand's closing movement, aligning with the action of the visual hand on the display. These findings may highlight the value of incorporating multimodal systems to boost the sense of ownership over the visual hand, potentially influencing both cognitive science and the field of stroke rehabilitation.

### Limitations

Future research should investigate the integration of visual information with an active grasping motion given that the scope of this study was constrained by its exclusive emphasis on passive motion. Extending the study's scope through the inclusion of a larger and more diverse sample size would enhance its comprehensiveness. For future research, it would be preferable to introduce a third condition in which the real and seen hands are placed in two different positions to enable isolation of the SoO from SoA. Our study lacks an additional experiment to quantitatively assess perceived pressure on the hand. The absence of psychophysics or subjective ratings for validation represents a potential limitation and an avenue for future research.

## Methods

### Participants

Twenty-three healthy participants (seven men and 16 women; average age, 30 ± 4.823 years), of which 21 were right-handed, were recruited with written informed consent. The required sample size for this study was 23 individuals, which was estimated based on a prior power analysis using a repeated-measures analysis of variance test within factors (significance level, α = 0.05; power β = 0.80; and medium effect size, f = 0.25). The experiment was approved by the ethics committee of the University of Reading (No. SBS 20-21 03) and performed according to the relevant guidelines and regulations.

### Experimental setup and procedures

**Figure 4:**
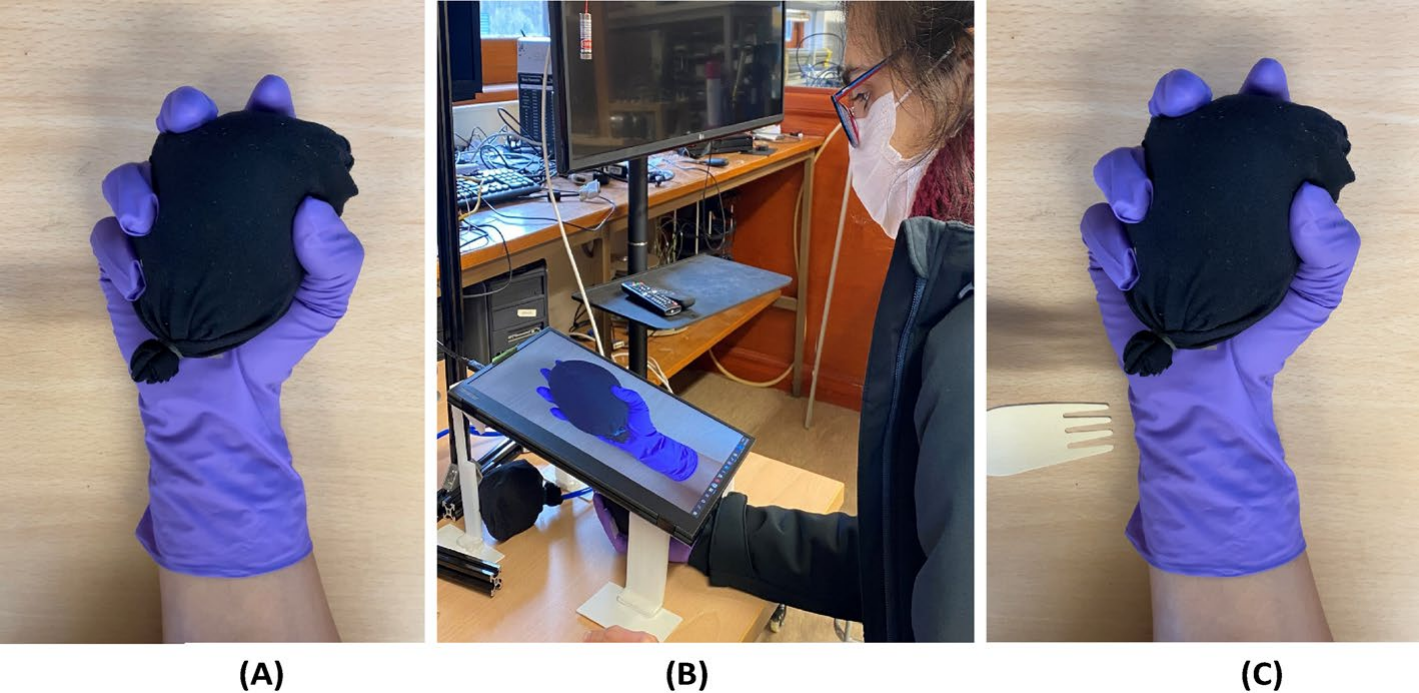
Experimental setup. (**A**) Image of a gloved hand holding an inflated balloon. (**B**) Image of the real hand beneath the display while the participant watches the video. (**C**) Image of a wooden fork approaching the real hand.

We designed and developed an integrated system that consisted of three set of feedback visual, kinesthetic, and haptic feedback (Figure 4). *Visual feedback* involved a pre-recorded video depicting a real hand on a display. The virtual hand held a balloon that was inflated and deflated. The real hand, positioned beneath the display, simultaneously held a balloon that was inflated/deflated to generate *kinesthetic movement* of the real hand and *haptic sensation* in the palm, as a form of pressure on the skin.

To minimize spatial discrepancy between the real (dominant) and virtual hand, both were covered in a purple glove, holding a balloon (Fig. 4A,B). To measure the extent of SoO, reaction times (RT) of participants to the sense of threat were measured.

To measure the RT, the participants were asked to use the index finger of their other hand to press the space bar on a keyboard as quickly as possible when they felt threatened by the virtual fork coming down upon the virtual hand (Figure 4C).

## Detailed explanation of feedback (visual, haptic, and kinesthetics) and conditions

**Figure 5:**
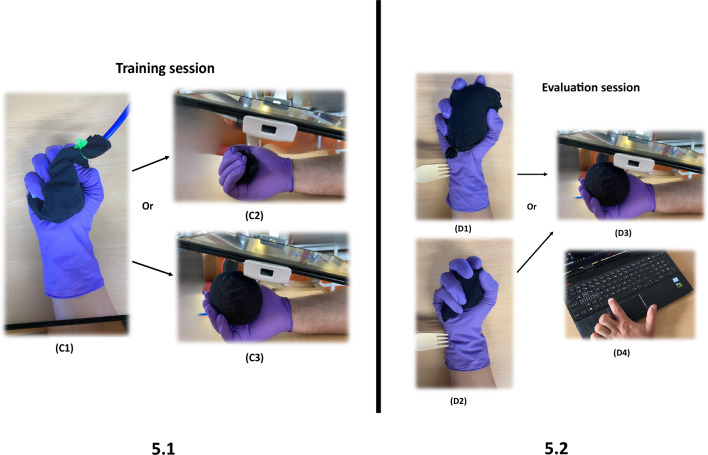
Flow of the experiment.

We tested two conditions where, in both, three sets of feedback (visual, haptic, and kinesthetic) were synchronized (Fig. 5). In the first condition, the three sets of feedback were “in-phase” [Fig. 5C1,C2], while in the second condition, they were in “anti-phase” [Fig. 5C1,C3]. Both conditions demonstrated the correlation of kinesthetic/haptic feedback induced by the balloon with the motion of the virtual hand on the display.

*Under the first condition “in-phase”, *the virtual hand attached to the virtual balloon opened when the balloon inflated on the display. Underneath the display, the participant’s real hand also opened as the real balloon inflated. The same relation applied in the case of balloon deflation and the resultant closing of the hand. The visual information of opening/closing of the hand was repeatedly synchronized with the inflation/deflation of the balloon in the same phase [Fig. 5C1,C2]. As the participant relaxes the hand, the pressure of the balloon can open up the hand, i.e., the fingers are extending, and when the balloon is deflating, the fingers will naturally follow the curvature of the balloon surface to close the hand. Thus, as a result of the synchronous in-phase repetition of visual information and the inflation–deflation cycle of the balloon, haptic feedback in the form of pressure on the skin and the corresponding kinesthetic feedback (flexing/extending of fingers) will be generated. On the contrary, *under the second condition, these two actions were in “anti-phase”,* when the virtual hand closed on the display, the balloon inflated, and the real hand opened underneath the display because the hand was attached to the balloon surface [Fig. 5C1,C3]. In both conditions, the kinesthetic and haptic feedback received by the real hand from the inflating/ deflating balloon were in synchrony.

### The training session

The three types of feedback are synchronous in the in-phase condition, represented as (C1 and C2). Under the anti-phase condition, it is represented as (C1 and C3).

### The evaluation session

Under in-phase condition, (D1 and D3), both balloons were inflated. Under anti-phase condition (D2 and D3) the balloon in the display is deflated, whereas the real balloon is inflated. Under both conditions, a virtual fork is moving towards the virtual hand and D4 represents the other hand while pressing on the keyboard.

### Measurement of the reaction time

As stated earlier, the assessment of SoO has conventionally relied on subjective measures such as questionnaires^45,46^. This experiment emphasises the use of objective measures to determine SoO rather than subjective techniques.

The utilization of reaction time (RT) has been recently demonstrated by Aizu et al. (2018 and 2022). In their studies, participants were instructed to switch off a light that was projected onto an extended part of the body. A shorter RT was indicative of a greater SoO.

Our method for measuring RT is a simple extension of this approach against a perceived threat. The level of fear is reportedly associated with the SoO over a fake hand^17^. We followed their work and examined participants’ RT to a threat as a measure of their SoO, with a shorter RT corresponding to a higher SoO.

### Experimental protocol for training and evaluation sessions

The experiment was performed under two conditions (Figs. 1 and 5), each comprising three training sessions followed by evaluation sessions, in which four responses were recorded.

In both the sessions, the participants’ real hand holding the balloon was placed beneath the display. *During the training session*, the display showed a video depicting the dynamic movement of the balloon and the virtual hand, wherein the balloon was continuously deflating and inflating, and the virtual hand was continuously closing and opening (Fig. 5). Each training session comprised eight repetitions of inflation and deflation. One cycle generally required 140 ms (70 ms to full inflation and 70 ms to full deflation). Therefore, one training session was completed in 1 min and 120 ms (Fig. 1).

*During the evaluation session*, the display showed a video depicting a static image of the virtual hand holding the balloon (there was no movement of the virtual hand; it remained only in one position), either closed or opened, and a fork moving toward it (Fig. 5). The participants were instructed to quickly stop the fork's motion by pressing the keyboard's space bar once they perceived a threat (Fig. 5D4). Overall, 12 responses were recorded for each participant under each condition: four RTs after each training session for a total of three training sessions (Fig. 1). The conditions used in the evaluation and types of feedback are summarized in Tables 1 and 2. The entire session generally took 30–40 min to complete.

**Table 1:** Experimental protocol for conducting training and evaluation sessions.

Two sessions	Training session	Evaluation session
Details of sessions	Participants watched the video of virtual hand holding a balloon that can be inflated and deflated (depending on conditions in Table 2), while the real hand is underneath the display holding a dynamic balloon (Fig. 4)	Participants watched a static picture of a virtual hand holding a balloon inflated or deflated (depending on conditions as in Table 2), while the real hand underneath the display holding a static balloon (Fig. 4)

**Table 2:** This table provides an illustrative example of the evaluation session, encompassing both conditions and three set of feedback.

Feedback	In-phase condition	Anti-phase condition
Visual feedback	Hand closed (flex)	Hand closed (flex)
Haptic feedback	Balloon deflated	Balloon inflated
Kinesthetic feedback	Fingers flexed	Fingers extended

We hypothesize that when the balloon is inflated, there might be high pressure from the balloon on the skin, and vice versa.

Subsequently, the participants were asked to complete a questionnaire based on the design of the virtual haptic system, which had collected data regarding the SoO over the virtual hand. The response scale ranged from 0 (strongly disagree) to 5 (strongly agree). They were also asked to provide feedback about the sessions (Table 3).

**Table 3:** Participant’s questionnaire.

To what extent do you think that you wanted to stop the fork from reaching your hand as fast as you could in the *first condition*?”	
To what extent do you think that you wanted to stop the fork from reaching your hand as fast as you could in the *second condition*?”	
To what extent do you think that you are threatened and stressed from the fork while it is moving toward the virtual hand *first condition*?	
To what extent do you think that you are threatened and stressed from the fork while it is moving toward the virtual hand *second condition*?	
To what extent do you think that the virtual hand belonged to your body (was part of your body) in first condition?	
To what extent do you think that the virtual hand belonged to your body (was part of your body) in second condition?	

### Ethics approval and consent to participate

The experiment was approved by the ethical committee of the University of Reading (No. SBS 20-21 03) and performed according to the relevant guidelines and regulations.

### Supplementary Information

Supplementary Information.

## Data Availability

Datasets generated for this study are available upon request from the corresponding author.
