# Supplementary material for: Enhancement of sense of ownership using virtual and haptic feedback
Source: Sci Rep. 2024 Mar 1;14:5140. doi: 10.1038/s41598-024-55162-x (PMC10907564; doi:10.1038/s41598-024-55162-x)
Supplement: Supplementary file 1 — Supplementary Information. [file 41598_2024_55162_MOESM1_ESM.docx]

**Supplementary files**

The following table presents responses from all participants to six questions.

| ID/Question | Q1 | Q2 | Q3 | Q4 | Q5 | Q6 |
| --- | --- | --- | --- | --- | --- | --- |
| 1 | 3 | 2 | 4 | 2 | 4 | 2 |
| 2 | 1 | 4 | 2 | 5 | 0 | 5 |
| 3 | 0 | 5 | 0 | 5 | 0 | 5 |
| 4 | 2 | 4 | 1 | 5 | 1 | 5 |
| 5 | 3 | 5 | 1 | 3 | 1 | 5 |
| 6 | 2 | 5 | 2 | 5 | 1 | 5 |
| 7 | 4 | 3 | 5 | 2 | 5 | 2 |
| 8 | 2 | 4 | 0 | 5 | 2 | 5 |
| 9 | 0 | 5 | 0 | 4 | 0 | 5 |
| 10 | 0 | 4 | 1 | 5 | 0 | 5 |
| 11 | 0 | 5 | 1 | 5 | 1 | 5 |
| 12 | 1 | 5 | 1 | 5 | 1 | 5 |
| 13 | 1 | 4 | 1 | 5 | 0 | 5 |
| 14 | 2 | 5 | 1 | 5 | 0 | 5 |
| 15 | 2 | 5 | 1 | 5 | 1 | 5 |
| 16 | 1 | 5 | 1 | 5 | 0 | 5 |
| 17 | 2 | 4 | 1 | 5 | 1 | 5 |
| 18 | 5 | 2 | 3 | 1 | 5 | 2 |
| 19 | 0 | 5 | 1 | 5 | 1 | 5 |
| 20 | 0 | 4 | 1 | 5 | 1 | 5 |
| 21 | 3 | 5 | 0 | 5 | 0 | 5 |
| 22 | 1 | 4 | 1 | 5 | 0 | 5 |
| 23 | 2 | 5 | 2 | 5 | 0 | 5 |

**The experimental apparatus**

**Balloon movement**

The movement of the real balloon beneath the display was regulated using a single 12 V pump equipped with two ports: one for suction and the other for blowing air. These pump ports were connected to three 12 V solenoid valves, providing control over the airflow. This setup included three valves to manage the inflow and outflow of air from our system. This configuration enabled control over whether air could be drawn into our system from the outside (inflation) or expelled while preventing outside air from entering (deflation). The output from these valves was channeled into the balloon via plastic/silicone tubes. In terms of the electric signals: The examiner initiated the inflation/deflation processes by pressing a button, controlling the cycles. Each cycle achieved full inflation within 70 ms and full deflation within 70 ms, constituting one complete inflation/deflation cycle. This sequence was repeated eight times. The button was connected to an Arduino microcontroller, acting as the central processing unit. Upon button press, the microcontroller would register the signal and send an output signal to the relays. The relays functioned akin to switches, receiving the low-voltage signal (approximately 5 V) from the Arduino, then delivering 12 V from a power supply unit to activate both the pump and valves. These relays served as a safety measure, given the Arduino's limited capacity to handle only 5-8 V.

**Recording of participant responses**

There were a total of 12 reaction times observed for each condition, encompassing 12 reactions within the first condition and an equivalent 12 reactions within the second condition. After the training session, where the participant watched a video depicting the real hand holding a dynamic balloon on the display, involving 8 cycles of inflation and deflation, the first evaluation session followed. During each evaluation session, 4 responses were recorded for every participant, amounting to a total of three evaluation sessions.

During the evaluation session, as the video began, a static hand was shown holding either an inflated or deflated balloon, depending on the condition. Simultaneously, a fork approached the visual hand, and participants were instructed to stop the fork when feeling threatened. Participants stopping the fork's motion marked the end of the video, and the examiner recorded the exact time in an Excel file.
